# CAN YOU HEAR ME? IMPLICATIONS OF HEARING LOSS FOR SOCIAL TIES AMONG OLDER ADULTS

**DOI:** 10.1093/geroni/igad104.3206

**Published:** 2023-12-21

**Authors:** Kira Birditt, Angela Turkelson, Emily Noyer, Jane Stephenson, Karen Fingerman, Michael McKee

**Affiliations:** University of Michigan, Ann Arbor, Michigan, United States; University of Michigan, Ann Arbor, Michigan, United States; University of Michigan, Ann Arbor, Michigan, United States; University of Michigan, Ann Arbor, Michigan, United States; The University of Texas at Austin, Austin, Texas, United States; University of Michigan, Ann Arbor, Michigan, United States

## Abstract

Hearing loss is a leading source of disability among older adults; over 65% of those aged 71 and older have hearing loss. Hearing loss may be associated with reduced relationship quality due to greater communication challenges, conflict, and avoidance of social interactions. This study examined links between hearing loss and social ties among older people. Participants included 11,161 respondents from the 2016 and 2018 waves of the Health and Retirement Study (ages 50+) who completed a hearing test and reported on their social ties (contact, quality), social activities (e.g., charity work), and loneliness. Multilevel models controlling for age, gender, and education revealed that having hearing loss was associated with having lower positive quality and higher negative quality relationships with spouse and friends, higher negative relationship quality with children, having fewer close friends, meeting up less with friends, greater loneliness, doing less charity work, and less attendance at social and community events. Older age was associated with more detrimental outcomes. Older aged individuals with hearing loss met up with friends less often, talked on the phone less with friends, did fewer activities with grandchildren/extended children and neighborhood children, did less charity work, and attended fewer social events compared to younger individuals with hearing loss. Overall, older adults with hearing loss are at high risk for relationship problems and social isolation; especially as they grow older. Hearing loss and social ties are important targets for future interventions for older adults with hearing loss as they may be particularly vulnerable.

